# Modeling of electrohydrodynamic drying process using response surface methodology

**DOI:** 10.1002/fsn3.96

**Published:** 2014-02-28

**Authors:** Mohammad Jafar Dalvand, Seyed Saeid Mohtasebi, Shahin Rafiee

**Affiliations:** Department of Agricultural Machinery, Faculty of Agricultural Engineering and Technology, University of TehranKaraj, Iran

**Keywords:** Box–Behnken, EHD drying, energy consumption, response surface methodology, solar energy

## Abstract

Energy consumption index is one of the most important criteria for judging about new, and emerging drying technologies. One of such novel and promising alternative of drying process is called electrohydrodynamic (EHD) drying. In this work, a solar energy was used to maintain required energy of EHD drying process. Moreover, response surface methodology (RSM) was used to build a predictive model in order to investigate the combined effects of independent variables such as applied voltage, field strength, number of discharge electrode (needle), and air velocity on moisture ratio, energy efficiency, and energy consumption as responses of EHD drying process. Three-levels and four-factor Box–Behnken design was employed to evaluate the effects of independent variables on system responses. A stepwise approach was followed to build up a model that can map the entire response surface. The interior relationships between parameters were well defined by RSM.

## Introduction

**Figure 1:**
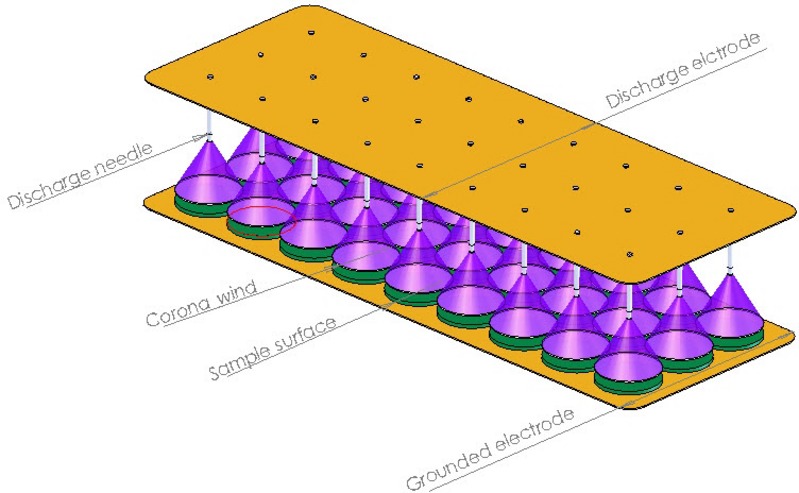
The mechanism of a corona wind.

Increasing global population and changes in the world economy are placing severe pressure on the utilization of natural resources for energy production. Moreover, emission of greenhouse gases from conventional dryers utilizing fossil fuels for operation in food industries has risen at both consumer and environmental concerns. Resolution of these issues requires innovative measures and practices for the development of alternative drying methods that have minimal or no effect on the food quality and also answer the environmental concerns associated with energy intensive drying processes (Bajgai et al. 2006; Singh et al. 2012). Therefore, energy consumption index is one of the most important criteria for judging new and emerging drying technologies (Bai et al. 2012). One of such novel and promising alternative drying process is called electrohydrodynamic (EHD) drying. EHD is a method of inducing electric wind that is generated by gaseous ions under the influence of a high-voltage electric field (Singh et al. 2012). A mechanism of an electric (corona) wind is presented in Figure 1. EHD drying method requires a simpler system and lower energy consumption when compared to a convective or freeze drying (Lai and Sharma 2005; Goodenough et al. 2007; Bai et al. 2011). The energy effectiveness of an EHD drying has been documented by Lai and Wong (2003), Balcer and Lai (2004). However, the product quality dried by an EHD dryer is not as good as that one obtained by a freeze dryer.

Response surface methodology (RSM) has important application in the design, development, and formulation of new products, as well as in the improvement of existing product design. In general, RSM which includes factorial design and also regression analysis, helps to evaluate the effective factors and to build the models in order to study the interactions and to select the optimum conditions of a desirable response (De Coninck et al. 2000; Dutta et al. 2004). RSM such as fuzzy logic, artificial neural networks, and genetic algorithms are also empirical models which are widely used for modeling of food processing due to the complexity of the reactions and nonhomogeneous structure of food products. RSM defines the effect of the independent variables, alone or in combination, on the process. In addition to analyzing the effects of the independent variables, this experimental methodology generates a mathematical model that accurately describes the overall process (Namal Senanayake and Shahidi 2002). The effectiveness of RSM methodology in optimization of processing conditions in food technology from raw to final products has been documented in the literature (Madamba 2002; Gan et al. 2007; Iwe and Agiriga 2013) and it has been successfully applied for modeling and optimization problems of roasting process.

Before applying the RSM methodology, first, it is necessary to choose an experimental design that will define which experiment should be carried out in the experimental region being studied. There are some experimental matrices for this purpose. Experimental design for first-order models (e.g., factorial designs) can be used when the data set does not present any curvature (Hanrahan and Lu 2006). However, to estimate a response function with an experimental data that cannot be described by linear functions, experimental designs for quadratic response surfaces such as three-level factorial, Box–Behnken, central composite, and Doehlert designs should be used.

### Box–Behnken design

Box–Behnken designs (Box et al. 1978) are response surface designs, especially made based on only three-coded levels −1, 0, and +1, whereas central composite made based on five levels. They are formed by combining two-level factorial designs with incomplete block designs. This procedure creates designs with desirable statistical properties but, most importantly, with only a fraction of the experiments required for a three-level factorial. Because there are only three levels, the quadratic model is appropriate. The coefficients of the quadratic model may be calculated using standard regression techniques (Francis et al. 2003).

In this article, the effects of the applied voltage, field strength, number of discharge electrodes (needles), and air velocity on EHD drying process are investigated with the help of Box–Behnken design and by using RSM. Finally, RSM was used to study the interaction of significant physical parameters and then to find a fitting model.

## Materials and Methods

### Materials

Fresh kiwi fruits (cv. *Hayward*) were supplied from the local market. Samples were transported to the Physical Laboratory of Faculty of Agricultural Engineering and Technology, University of Tehran, Karaj, Iran. Moisture content was determined using the oven method (Standard 1998). At the end of each drying process, the samples were taken out and dried in the oven for 24 h at 65°C until constant weight. Weighing was performed on a digital balance, and then moisture content (w.b.) was calculated. Before drying, the kiwi fruits were cut into circular slices of 3.5–5.0 cm in diameter and 2.0 mm in thickness.

### Experimental setup

**Figure 2:**
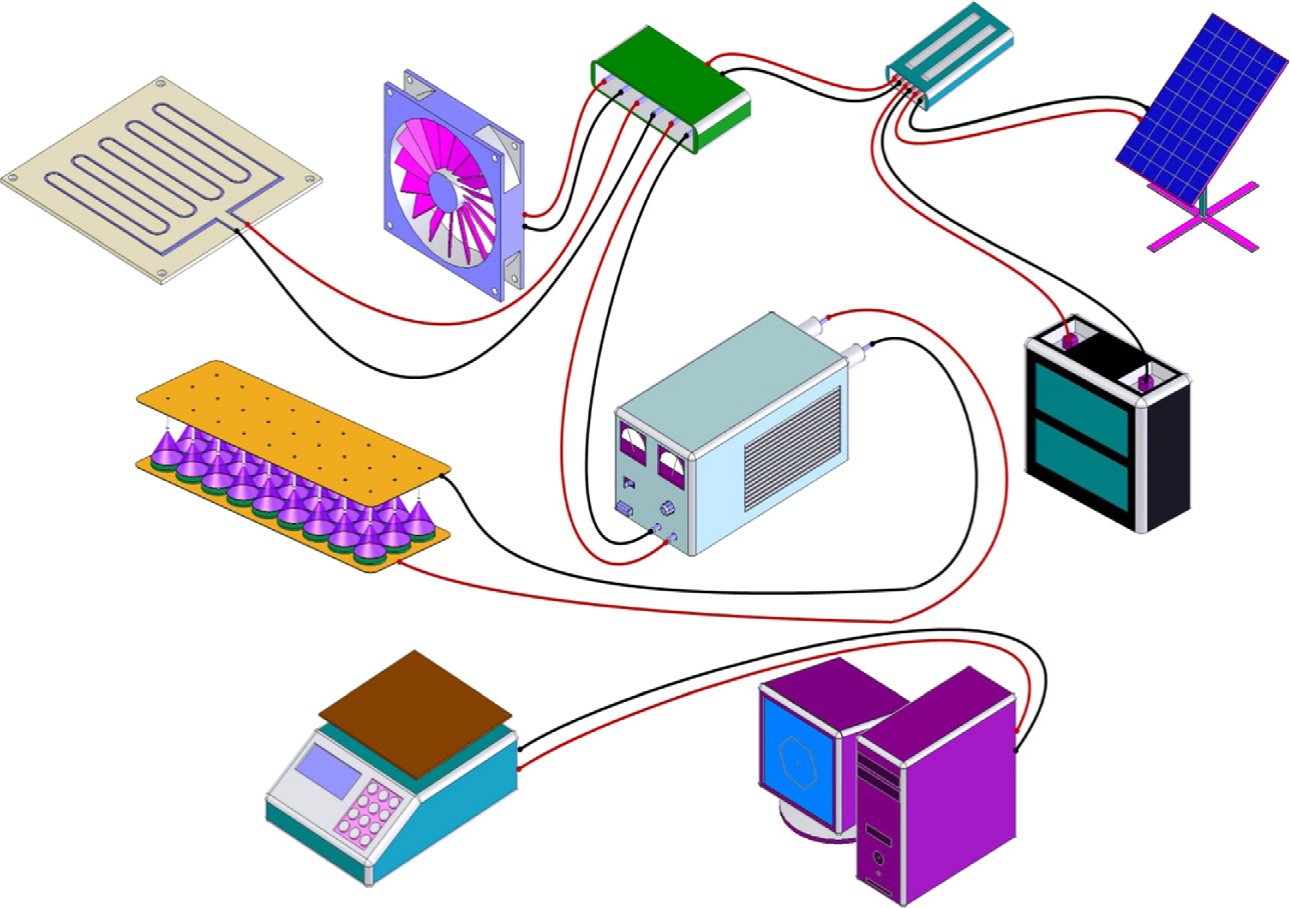
Components of a solar EHD dryer.

The experimental setup for a solar EHD drying is shown in Figure 2. It consists of an electric field apparatus supplied from a DC high-voltage generator which was designated and manufactured in Power Laboratory, with an output voltage of 0–30 kV and maximum current of 2 mA with both types of polarities. Other components are grounded plate electrode, discharge point electrode (needle), heater plate, solar power supply system, and some measurement instruments. The accuracy of the power supply was ±100 V for voltage and ±0.002 mA for current.

In the point-to-plane configuration, vertically mounted electrode with multiple pointed needles projected to a fixed horizontal grounded metallic plate on which the samples to be dried are placed. The distance between electrodes is adjustable between 0 and 8 cm. The sharp points of 17 needles (0.1 mm in point diameter) were fixedly connected to a direct current high-voltage power source that supplies a positive high voltage, vertically above the center of plane (25 × 28 cm) which was used as an electrically earthed reception plane. Electric field was applied to the samples by adjusting the high voltage and the electrode spacing. In order to change the desired high-voltage parameters for the EHD drying, a control circuit was added to the DC high-voltage generator. A solar power supply system consists of a 60 W photovoltaic module and an 18 Ah backup battery was considered for providing required energy of the system. Due to the fact that the earth rotates on its axis and orbits around the sun, if a photovoltaic module/cell is immobile, its absorption efficiency will be significantly less at certain times of the day and year. The photovoltaic module must be perpendicular to the sun for maximum solar absorption, which is done by using a tracking system. Thus, the photovoltaic module was equipped to a designated sun tracker. In this work, by changing the applied voltage, field strength, the number of discharge needles, and air velocity, the factors related to the drying process of thin-layer kiwi fruit such as moisture ratio (MR), energy efficiency, and energy consumption are investigated.

### Methods

The initial weight and temperature of the samples were measured and then samples were prepared for drying. Ambient conditions in the laboratory during EHD drying were 24°C and 20.8% relative humidity. The sample weight was measured at 10 min intervals. Each set of measurements was completed in less than 30 sec. During the entire experiment period, the change of ambient temperature was minimal since the laboratory was under well temperature control.

MR of kiwi during drying experiments was calculated using the following equation (Toğrul and Pehlivan 2003):


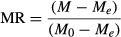
(1)

where *M*, *M*_*o*_, and *M*_*e*_ are moisture content at any drying time, initial moisture content, and equilibrium moisture content (kg water/kg dry matter), respectively. The values of *M*_*e*_ are relatively small compared to those of *M* or *M*_*o*_, hence the error involved in the simplification is negligible (Aghbashlo et al. 2008).

To evaluate energy consumption of the EHD drying process, the results are expressed in terms of consumed energy per unit of mass of moisture removed due to EHD effect as shown in the following equation:


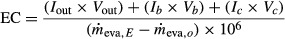
(2)

where EC is consumed energy in kJ/g, 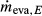 and 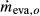 are the average rate of water evaporation due to phenomenon EHD-enhanced and control sample in kg/sec, respectively. *I*_out_ is consumed current by high-voltage electric field in ampere and *V*_out_ is applied voltage in volt. Subscripts “b” and “c” means blower and control circuit, respectively. At the end of each experiment, difference between amount of water evaporated due to phenomenon EHD enhanced and similar value for the control sample was calculated. The energy consumption per unit of mass of moisture removed was obtained by dividing consumed energy by all components during experiment per this value.

In order to calculate the energy efficiency of high-voltage power supply, voltage–current input and output were measured during experiments. Then using equation (3) efficiency of high-voltage power supply was obtained:


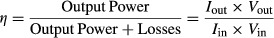
(3)

where *I* is current in Ampere and *V* is applied voltage in Volt, in this equation, the index “in” means input and “out” means output from the power supply.

### Experimental design and RSM

In order to examine the effects of combinations of the four different factors (independent variables) consist of air velocity, applied voltage, field strength, and number of needles on parameters of the EHD drying process of kiwi fruit (MR, energy consumption, and energy efficiency) and derive a fitting model, RSM was used. A four-factor and three-coded level (−1, 0, and +1) Box–Behnken design was used to study the interactions of significant physical parameters and later on to find fitting models for EHD drying process. The factors levels with the corresponding real values are shown in Table 1.

**Table 1:** Levels of the independent variables

			Range and levels		
			
Independent variable	Type	Symbol	−1	0	1
Air velocity (msec^−1^)	Numerical	*X*_1_	0	0.2	0.4
Applied voltage (kV)	Numerical	*X*_2_	6	10.5	15
Field strength (kV cm^−1^)	Numerical	*X*_3_	3	4.5	6
Number of needles	Categorical	*X*_4_	1	9	17

The chosen independent variables used in this study were coded according to the following equation:


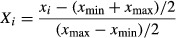
(4)

where *X*_*i*_ is a dimensionless coded value of the variable *x*_*i*_, and *x*_max_, and *x*_min_ are the maximum and minimum values of the natural variables, respectively. The behavior of the system is explained by the following empirical polynomial model equations:


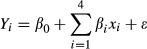
(5)


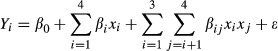
(6)


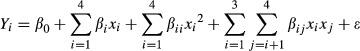
(7)

where *Y* is the predicted response, *β*_0_ is the intercept term, *β*_*i*_s are the linear coefficients, *β*_*ii*_s are the quadratic coefficients, *β*_*ij*_s are the interaction coefficients, *x*_*i*_s and *x*_*j*_s are input variables which affect the response *Y*, *x*_*i*_*x*_*j*_s are the interaction effect, and *ε* is a random error (Aksu et al. 2002; Göksungur et al. 2005; Aksu and Gönen 2006).

The predicted polynomial model was analyzed using the response surface regression procedure. The coefficients of the equations (4–6) and graphical analysis of the obtained data were determined using an algorithm written in Design Expert 8 (Stat-Ease Inc., Minneapolis, MN) software. There being six replicates at the center points and single runs for each of the other combinations, 30 runs were done in a totally random order. Duplicate experiments were carried out at all design points. As usual, the experiments were performed in random order to avoid systematic error. The design matrix based on Box–Behnken design is shown in Table 2.

**Table 2:** Design matrix processed by Design Expert 8

	Applied voltage		Field strength		Air velocity		Number of needles	
				
Run order	Coded	Real	Coded	Real	Coded	Real	Coded	Real
1	−1	6	−1	3	0	0.2	0	9
2	−1	6	0	4.5	−1	0	0	9
3	−1	6	0	4.5	0	0.2	−1	1
4	−1	6	0	4.5	0	0.2	1	17
5	−1	6	0	4.5	1	0.4	0	9
6	−1	6	1	6	0	0.2	0	9
7	0	10.5	−1	3	1	0.4	0	9
8	0	10.5	−1	3	0	0.2	1	17
9	0	10.5	−1	3	0	0.2	−1	1
10	0	10.5	−1	3	1	0.4	0	9
11	0	10.5	0	4.5	−1	0	1	17
12	0	10.5	0	4.5	−1	0	−1	1
13	0	10.5	0	4.5	0	0.2	0	9
14	0	10.5	0	4.5	0	0.2	0	9
15	0	10.5	0	4.5	0	0.2	0	9
16	0	10.5	0	4.5	0	0.2	0	9
17	0	10.5	0	4.5	0	0.2	0	9
18	0	10.5	0	4.5	0	0.2	0	9
19	0	10.5	0	4.5	1	0.4	−1	1
20	0	10.5	0	4.5	1	0.4	1	17
21	0	10.5	1	6	−1	0	0	9
22	0	10.5	1	6	0	0.2	−1	1
23	0	10.5	1	6	0	0.2	1	17
24	0	10.5	1	6	1	0.4	0	9
25	1	15	−1	3	0	0.2	0	9
26	1	15	0	4.5	−1	0	0	9
27	1	15	0	4.5	0	0.2	−1	1
28	1	15	0	4.5	0	0.2	1	17
29	1	15	0	4.5	1	0.4	0	9
30	1	15	1	6	0	0.2	0	9

### Measures of model adequacy

As in the case of a simple linear regression, analysis of a fitted multiple linear regression model is important before inferences based on the model are undertaken. This section presents some techniques that can be used to evaluate the appropriateness of the multiple linear regression model. The coefficient of multiple determination is defined as follows:


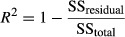
(8)

where SS_residual_ is sum of squares of the differences between the predicted and actual values and SS_total_ is sum of squares of the differences between the predicted and average of actual values. The value of *R*^2^ increases as more terms are added to the model, even if the new term does not contribute significantly to the model. A better statistic to use is the adjusted *R*^2^ statistic defined as follows:


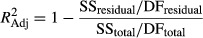
(9)

where DF_residual_ and DF_total_ are residual and total degrees of freedom, respectively. The adjusted *R*^2^ only increases when significant terms are added to the model. Other values which were used along with these values are RMSE (Root Mean Square Error), PRESS (prediction error sum of squares) and 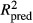. RMSE is presented in the following equation:


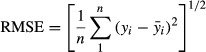
(10)

where *y*_*i*_ is the *i*th value of the variable to be predicted and 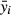 is the predicted value corresponding to *y*_*i*_. PRESS is an abbreviation for prediction error sum of squares and defined by following equation:


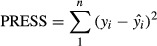
(11)

where *y*_*i*_ is the *i*th value of the variable to be predicted and 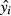 is the predicted value based on a model that contains all observed values except the *y*_*i*_ 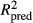 referred as a prediction *R*^2^, is obtained using PRESS as shown next:


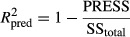
(12)

The values of *R*^2^, 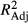 and RMSE are indicators of how well the regression model fits the observed data. On the other hand, the values of PRESS and 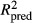 are indicators of how well the regression model predicts new observations. The higher values of PRESS or lower values of 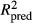 indicate a model that predicts poorly. On the other hand, the prediction of *R*^2^ and the adjusted *R*^2^ should be within 0.20 of each other. Otherwise, there may be a problem with either the data or the model. Different transformations or a different order polynomial have been suggested to overcome this problem (Myers Raymond and Montgomery 2002). As we know, most important object of using design of experiment is to predict unknown condition. Therefore, the best model was selected based on higher value of 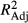, 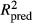 and lower in PRESS. Such values indicate that the regression model has fitted the data well and also has predicted predicts well.

## Results and Discussion

### MR response

In general, variation of MR in high-voltage field strength is influenced by several parameters such as applied voltage, field strength, air velocity, and number of discharge needles among others, and their effects may be either independent or interactive. However, the influence of the mention parameters on EHD drying process of kiwi fruit has not been reported yet. We followed a stepwise approach to build up the model that can map the entire response surface. Besides, the polynomial models fitted to the data, special quadratic model was also statistically significant. Hence, we used the special quadratic model to interpret trend of responses and to generate the contour and three-dimensional (3D) plots. Reduced quadratic model was obtained by eliminating terms which were not significant in full quadratic model. Therefore, in this model, all the coefficients were significant (*P* < 0.01). The evaluation indices of multiple regression obtained from a least squares analysis used to predict a polynomial model for MR estimation are summarized in Table 3.

**Table 3:** Adequacy of models for moisture ratio

	Statistical index				
	
Model	*R*^2^	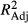	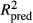	RMSE	PRESS
Linear	0.718	0.637	0.560	0.038	0.056
2FI	0.735	0.595	0.128	0.042	0.110
Quadratic	0.903	0.813	0.453	0.029	0.070
Red. quadratic	**0.884**	**0.864**	**0.826**	**0.024**	**0.025**
Cubic	0.974	0.893	0.012	0.022	0.430

Moisture content data obtained at different experiments were converted to MR and then fitted to appropriate polynomial models. Table 3 illustrates the fitting results (*R*^2^, 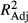, 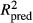, RMSE, and PRESS) for the models using the experimental data, the best-fitting model and are shown in black bolded. The criterion for selection of the best model describing variation of MR was the model with the highest *R*^2^ and 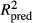, and the lowest PRESS values.

According to the results presented in Table 3, for the MR estimation during drying process based on applied voltage, field strength, air velocity, and number of discharge needle, the reduced quadratic model was the best model with *R*^2^ = 0.884 and following equation:


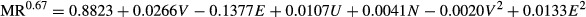


**Figure 3:**
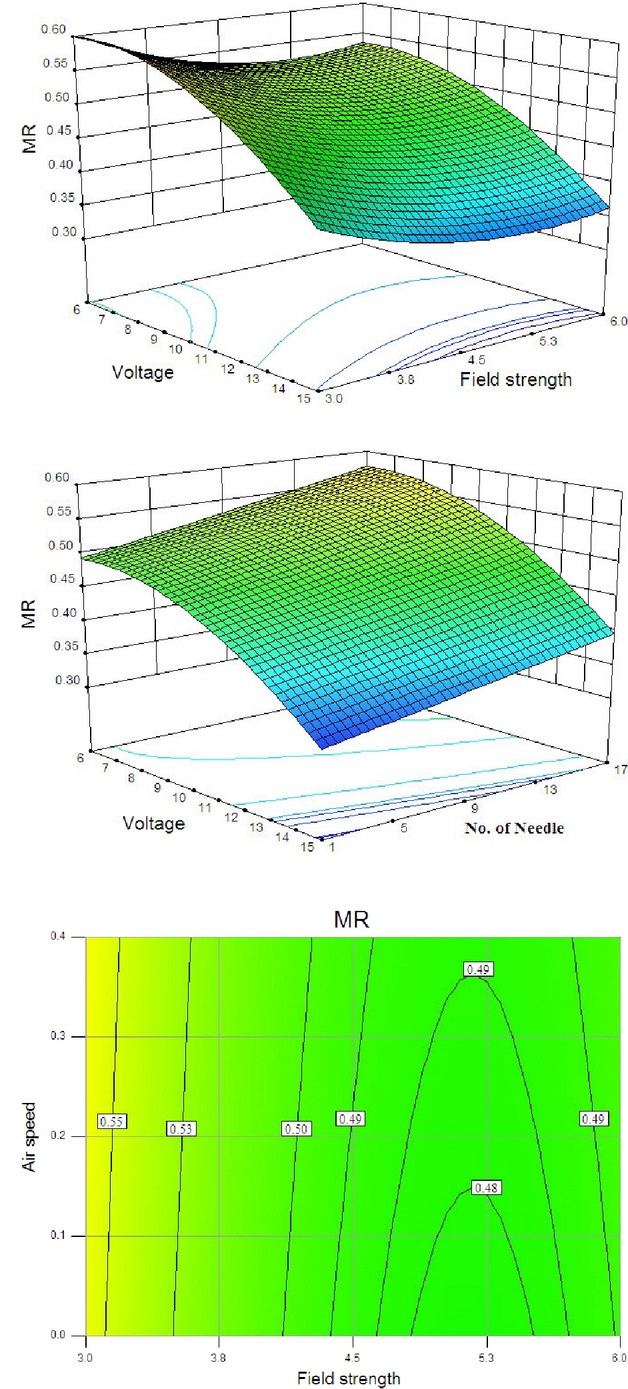
Response surface for the moisture ratio (MR) as a function of (A) applied voltage and field strength and (B) applied voltage and number of discharge needle and (C) contour plot for the MR as a function of air velocity and field strength.

Although Table 3 shows higher value of *R*^2^ for full quadratic and cubic models, the difference between their 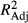 and 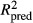, are greater than 0.2. Hence, these models cannot well describe the experimental data. This predicted polynomial model was used to obtain 3D response surface and contour plot for all interactions (Fig. 3). Other independent variables were kept at the middle level to obtain these figures.

It is clear from Figure 3A that the moisture content decreases faster in higher drying voltage. This means that the higher the voltage between the two electrodes, the stronger electric field strengths will be, and, the faster drying rate is, as a result, the moisture content decreases faster. The enhancement in mass transfer rate could be attributed to the electric wind induced by EHD as the main driving force (Chaktranond and Rattanadecho 2010). Bai et al. (2011) investigated the influence of operating parameters on energy consumption of EHD dryer of tofu. They reported that the drying rate was significantly improved with drying voltage of 45 kV, and the drying rate of tofu samples was almost eight times as much as that of control at the same temperature (Bai et al. 2011). Figure 3B and C shows that increase in number of discharge needle leads to decrease in drying rate and consequently MR increases. However, the number of discharge needle showed a more significant effect on MR with respect to the air velocity.

### Energy efficiency response

According to the results which are shown in Table 4, for prediction of the energy efficiency of high-voltage power supply during drying process based on applied voltage, field strength, air velocity, and number of discharge needle, the reduced quadratic model was the best model with *R*^2^ = 0.987 and following equation:


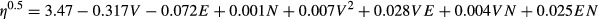


**Table 4:** Adequacy of the model for efficiency

	Statistical index				
	
Model	*R*^2^	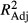	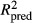	RMSE	PRESS
Linear	0.958	0.951	0.935	0.21	1.65
2FI	0.981	0.972	0.940	0.16	1.51
Quadratic	0.988	0.977	0.932	0.14	1.72
Red. quadratic	**0.987**	**0.982**	**0.969**	**0.12**	**0.99**
Cubic	0.999	0.995	0.835	0.061	3.39

The best-fitting model values are shown in bold.

Despite cubic and quadratic model being superior in the most indices, but they are not considered as a top or the best model. This choice is due to being superior in 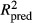 and PRESS. As discussed earlier, reducing the number of experiments was superior reliability of RSM, therefore deciding about the rest is more important and these indicators show the power estimation of the model for untested condition. Based on the equation, the interaction effect of applied voltage and filed strength, applied voltage and number of discharge needle, and finally field strength and number of discharge needle on efficiency is being significant, due to these parameters appeared in the equation of model.

**Figure 4:**
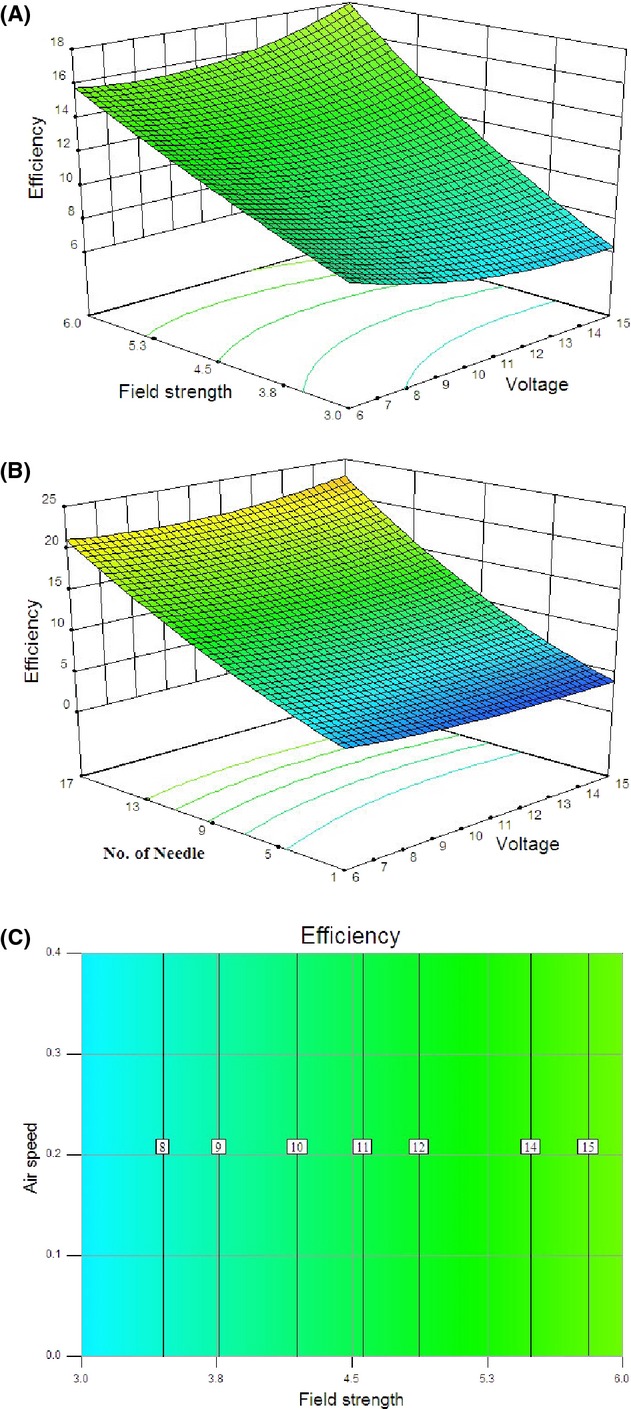
Response surface for the efficiency as a function of (A) applied voltage and field strength and (B) applied voltage and number of discharge needle and (C) contour plot for the efficiency as a function of air velocity and field strength.

Analyzing the contour plots for energy efficiency of high-voltage power supply during drying process was the best way to evaluate the relationships between responses, variables, and interactions that existed herein. The 3D response surfaces were plotted in Figure 4 as a function of the interactions of any two of the variables by holding the other one at its medium value. The entire relationships between independent variables and response can be better understood by examining the planned series of contour plots (Fig. 4) generated from the predicted model based on the model equation and by holding other variables in their constant values. Figure 4C revealed that air velocity did not show any significant effect on the energy efficiency of high-voltage power supply, whereas in Figure 4A and B number of discharge needles, field strength and applied voltage showed significant effect on the efficiency. Intensity of the air flow has an indirect impact on energy efficiency of high-voltage power supply. Variation in intensity of the air flow may lead to increase evaporation rate of the product and as a result consumed energy and energy efficiency may be changed. However, in this case, air velocity did not show any significant effect on the energy efficiency.

With the increase of applied voltage, field strength and number of discharge needle, we observed an increase in the value of efficiency. But, as seen in Figure 4A and B, applied voltage has a less effect on the efficiency as compared to other parameters. This could be due to a change in the output voltage of the power supply is lid to changing in the saturation conditions and according to equations mentioned in resources changing in saturation condition lead to different values of efficiency. Efficiency of a power supply reached to nearly 90% in its saturation condition. Figure 4C indicates the efficiency of a high-voltage power supply along with air velocity and field strength and it is clear that with increasing field strength, efficiency increases but almost there is no effect with increasing air velocity. Increasing the electric field strength increases energy consumption, and energy efficiency increased with increasing in consumed energy and this trend is followed up to saturation conditions.

### Energy consumption response

The results of the first, second, and the third order response surface models fitting are given in Table 5. To test fitness of the models, the regression equation, determination coefficient *R*^2^, 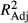, 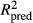, RMSE, and PRESS were evaluated. The coefficients of regression equation for energy consumption were calculated based on applied voltage, field strength, air velocity, and number of discharge needles using RSM procedure and then the following regression equation was obtained.

**Table 5:** Adequacy of the model for energy consumption

	Statistical index				
	
Model	*R*^2^	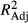	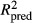	RMSE	PRESS
Linear	0.734	0.691	0.595	0.16	0.93
2FI	0.740	0.604	0.192	0.18	1.86
Quadratic	0.981	0.964	0.894	0.053	0.24
Red. quadratic	**0.974**	**0.965**	**0.946**	**0.052**	**0.12**
Cubic	0.998	0.994	0.751	0.021	0.37

The best-fitting model values are shown in bold.


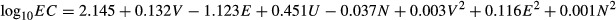


The model presented a high determination coefficient (*R*^2^ = 0.974) explaining 97% of the variability in the response (Table 5). The value of the adjusted determination coefficient (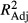 = 0.965) is also very high to indicate a high significance of the model (Khuri and Cornell 1996; Tanyildizi et al. 2005). The prediction determination coefficient of 0.946 indicates an adequacy of model for prediction objectives. The above equation showed that energy consumption of kiwi fruit during drying process have a complex relationship with independent variables that encompass both the first and second order polynomial and may have more than one maximum point.

**Figure 5:**
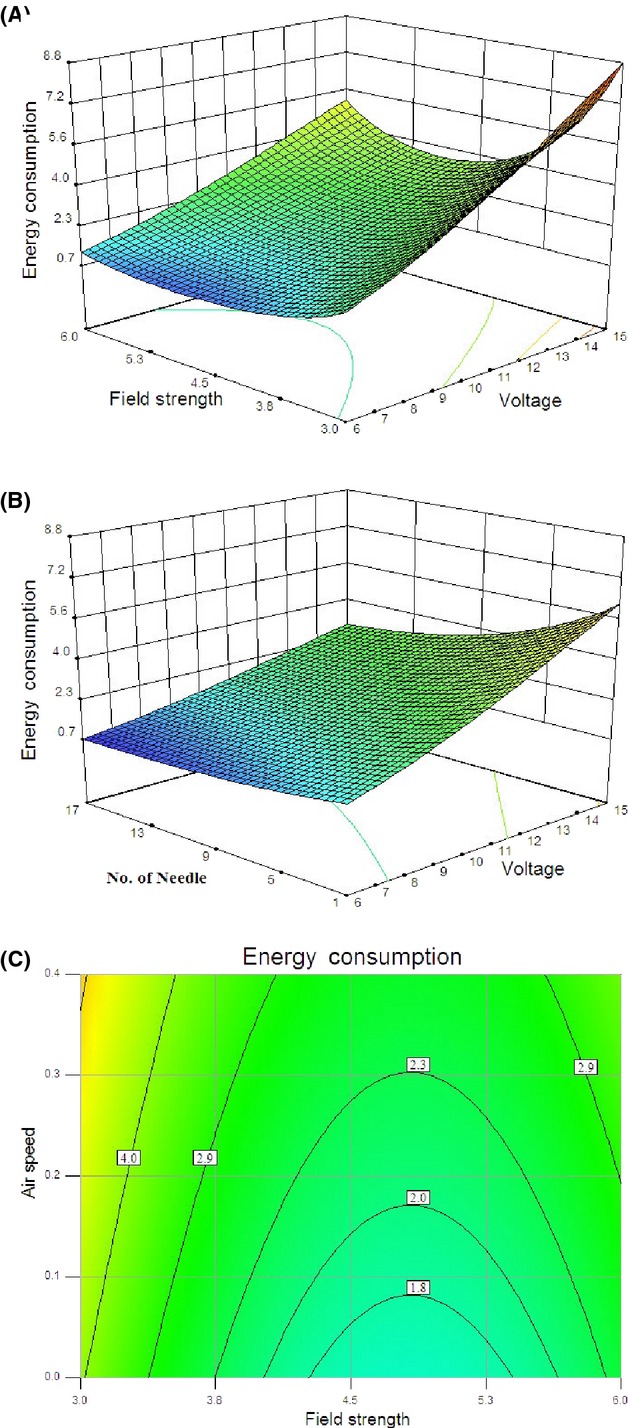
Response surface for the energy consumption as a function of (A) applied voltage and field strength and (B) applied voltage and number of discharge needle and (C) contour plot for the energy consumption as a function of air velocity and field strength.

The 3D response surfaces were plotted in Figure 5, as a function of the interactions of any two of the variables by holding the other one at its medium value. All plots in Figure 5 showed similar relationships with respect to the effects of each variable. The responses obtained had nonlinear nature suggesting that there were well-defined optimum operating conditions with respect to field strength. However, the concavity was not high enough, as the surfaces were rather symmetrical and a little flat near the optimum condition (field strength = 5.2 kV/cm). The variations of energy consumption at different applied voltages are given in Figure 5A. It can be seen that energy consumption for different field strength increases some nonlinearly with increasing in applied voltage.

As seen in Figure 5B, energy consumption decreases continuously with increase in number of discharge needle and reached to its minimum value in a combination of 17 discharge needles. With increasing the voltage, the energy consumption changes will tend to be nonlinear. The number of discharge needles can have quite conflicting effects depending on how they should get together. The counter plot curves for energy consumption as a function of field strength and number of discharge needles are plotted in Figure 5C. An increase in air velocity resulted in higher energy consumption in drying process, which revealed that high air velocity may lead to reduction of EHD performance and it also increases energy consumption per unit of water evaporation.

## Conclusion

The RSM based on four variables, Box–Behnken design was used to determine the effect of applied voltage (ranging 6–15 kV), field strength (ranging 3–6 kVcm^−1^), air velocity (ranging 0.0–0.4 msec^−1^), and number of discharge needles (ranging 1–17) on the MR, efficiency, and energy consumption. The regression analysis, statistical significance, and response surface were applied using Design Expert Software for forecasting the responses in all experimental areas. Models were developed to correlate variables to the responses. The results of this study leads to following conclusions:

1. In all cases, reduced quadratic model had the best fitness to experimental data.
2. With the increase of applied voltage and field strength (up to 5.2 kVcm^−1^), a decrease in the value of MR was observed.
3. Role of air velocity can be ignored but field strength was found to have the most significant effect on efficiency.
4. Increasing in field strength up to 5.2 kVcm^−1^ and decreasing in applied voltage lid to lower value of energy consumption.
